# Artificial Intelligence Analysis of Symmetry and Emotions in Facial Palsy Patients After Botulinum Toxin A Injections

**DOI:** 10.3390/toxins17120597

**Published:** 2025-12-15

**Authors:** Seraina L. C. Müller, Chantal Zeier, Pablo Pfister, Nadia Menzi, Bita Tafrishi, Dirk J. Schaefer, Jan A. Plock, Tarek Ismail, Holger J. Klein

**Affiliations:** 1 Department of Plastic, Reconstructive, Aesthetic, and Hand Surgery, University Hospital Basel, 4031 Basel, Switzerland; 2 Medical University of Zurich, 8006 Zurich, Switzerland; 3 Department of Plastic Surgery and Hand Surgery, Cantonal Hospital Aarau, 5001 Aarau, Switzerland

**Keywords:** Botulinum toxin, facial palsy, artificial intelligence, PROMs

## Abstract

Facial palsy affects millions worldwide. Botulinum toxin Type A (BoNT-A) is an established treatment for non-flaccid facial palsy, yet objective evidence remains limited. This study evaluates the effects of BoNT-A using AI-based tools and patient-reported outcome measures (PROMs). In this prospective observational study, patients with non-flaccid facial palsy received individualized BoNT-A injections. Exclusion criteria included age < 18, hypersensitivity to BoNT-A, or lack of follow-up. Assessments were conducted before and 3 weeks after treatment, including facial symmetry (Emotrics^®^), emotion expression (FaceReader™), and PROMs (FaCE and FDI). Eleven patients (mean age 50.1 ± 18 years) were included. BoNT-A significantly improved dynamic facial symmetry: eyebrow raising (*p* = 0.032), smile angle (*p* = 0.005), and lower lip height (*p* = 0.042). Emotion analysis showed no significant changes. PROMs revealed improvements in social well-being (FDI, *p* = 0.004) and aesthetic satisfaction (FaCE, *p* = 0.035), while functional FDI scores remained unchanged (*p* = 0.406). BoNT-A improves objective symmetry and patient satisfaction in non-flaccid facial palsy. The lack of change in emotional expression may reflect improved symmetry at the cost of dynamic muscle activation.

## 1. Introduction

Facial palsy, a lower motor neuron lesion, affects millions of people worldwide annually and can result from various causes, including infections, trauma, tumors, autoimmune disorders, and pregnancy [1]. The facial nerve plays a critical role in protection, aesthetics, and communication, and its dysfunction has widespread consequences [2]. Aesthetic concerns arise from facial asymmetry at rest, as well as during performing emotions and contributing to a negative self-image [3]. Numerous studies have shown that individuals with facial palsy frequently experience psychosocial issues such as depression, anxiety, and social withdrawal, which have a significant impact on an individual’s quality of life [4,5]. Approximately two-thirds of individuals affected by non-Bell’s palsy forms experience lasting impairments, primarily due to aberrant nerve regeneration [1]. This can manifest as non-flaccid facial palsy, characterized by involuntary, synchronous, or overactive facial muscle activity. Other clinical manifestations include facial muscle spasms or cramping, changes in the hearing threshold due to stapedial synkinesis, gustatory hyperlacrimation and gustatory sweating (Frey’s syndrome) [6]. Management options include non-surgical therapies such as biofeedback, Botulinum toxin Type A (BoNT-A) injections, or, in selected cases, surgical intervention such as myectomy or neurotomy [7]. Targeted injections of BoNT-A into hyperactive muscles reduce abnormal movements and can improve facial symmetry [8]. Injections may also be used on the non-paralyzed side to enhance overall facial balance [9]. Until now, most evaluation strategies for facial palsy have relied on subjective patient-reported outcome measures (PROMs), such as the well-known Facial Clinimetric Evaluation [10], Facial Disability Index [11] and FACE-Q [12]. Further well-known subjective physician-led scoring systems include the House–Brackmann Grading scale [13], Sunnybrook Facial Grading Scale [14], Sydney Facial Grading System [15], in addition to the newer eFACE, an app-based tool to grade facial paralysis [16]. Both PROMs and clinician-graded tools introduce a degree of subjectivity. The emergence of artificial intelligence (AI) introduces new possibilities for objective lay person assessment due to advances in facial point identification and emotion detection [17]. Emotrics^®^, is a tool designed to provide static objective assessments of facial palsy patients, and it is validated for the assessment of facial palsy [18,19]. FaceReader™, an AI-driven software tool trained in over 100,000 facial images and achieving very high precision levels, is used for analyzing spontaneous and evoked emotions such as joy, sadness, anger, surprise, disgust, fear, and neutrality [20]. It is used to assess nerve regeneration in face transplants and is currently validated for neutral and happy in facial palsy [21,22,23]. BoNT-A is a minimally invasive treatment with a well-established safety profile and significant therapeutic potential [24]. However, the literature remains scarce, particularly regarding objective outcome assessment and the use of standardized treatment protocols in facial palsy patients [25,26]. In this study, we present a novel approach by combining AI-based facial analysis with PROMs. Our aim is to evaluate the outcomes of BoNT-A injections using both objective AI tools and subjective patient-reported outcome measures (PROMs).

## 2. Results

### 2.1. Patient Cohort

A total of 11 patients were included. Of the study population, 10 were female, and 1 was male. The mean age was 50.1 years (SD 18, range 24.5 to 85 years). The median Body Mass Index (BMI) was 23.1 kg/m^2^ (IQR: Q1 22.3 and Q3 26.95, range 18 to 38.6 kg/m^2^). The causes of facial paralysis varied among the 11 participants. In 5 cases, the etiology was idiopathic. Three other patients were diagnosed with facial paralysis due to herpes zoster infection. One patient experienced iatrogenic paralysis. Another patient developed paralysis postpartum, and one patient’s facial paralysis was associated with a tumor. No cases of bilateral paralysis were reported. Six participants had previously undergone logotherapy. Additionally, two participants had undergone lip filler treatments, and another two had surgical interventions (static procedures), all over one year before the injection of BoNT-A. No complications were observed. Demographics and patient characteristics are outlined in Table 1.

### 2.2. Amount and Site of BoNT-A Injection

**Figure 1:**
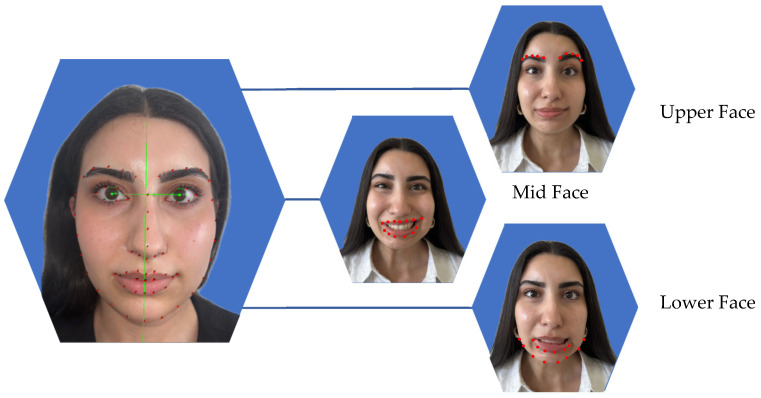
Facial symmetry analysis using Emotrics^®^ was performed in three facial regions. The green lines show the facial axis. Red dots indicating the area which was analyzed. In the upper face, symmetry was assessed at rest and during eyebrow elevation by measuring eyebrow height deviation (mm). In the midface, photographs at rest and during smiling with visible teeth were analyzed to evaluate the smile angle (degrees). For the lower face, images at rest and while showing the lower lip were used to measure lower lip height deviation (mm).

The mean total dose of BoNT-A administered was 21 IU (SD 9, range 3 to 34). On average, 18 IU (SD 7; range 11–24 IU) were injected into the affected side. Eight out of eleven patients (73%) received BoNT-A injections in the upper face, with a median dose of 3.0 IU (IQR1 2.75, IQR3 4.0, min to max 1–9 IU). Of the total dose administered in this region, 75% was delivered to the eyebrow area. In the midface, 10 patients (91%) received injections, with a median dose of 4.0 IU (IQR1 2.25, IQR3 4.75, min to max 2–10 IU). In the lower face and neck region, all patients (100%) received BoNT-A, with a median dose of 10.0 IU (IQR1 8.0, IQR3 13.0, min to max 2–20 IU) (Figure 1).

### 2.3. Facial Symmetry, Emotrics^®^

**Figure 2:**
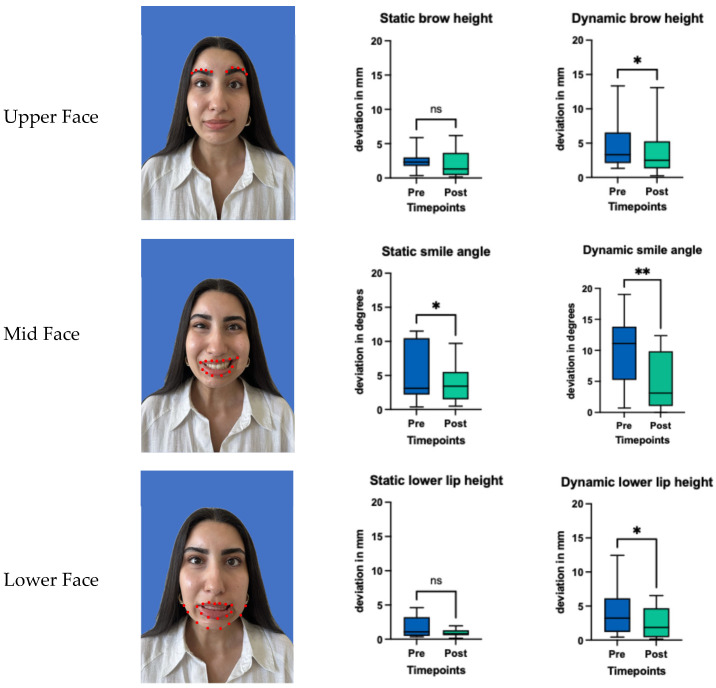
Facial symmetry analysis using Emotrics^®^ in static and dynamic states before and after (3 weeks) BoNT-A treatment. Red dots indicating the area which was analyzed. Boxplots represent the mean score, with whiskers indicating the minimum and maximum values. Statistical significance was assessed using a Wilcoxon matched pairs signed rank test, with a significance threshold set at * = *p* < 0.05. ** = *p* < 0.005 and ns = non-significant.

Facial symmetry was evaluated across the upper, mid, and lower facial regions using Emotrics^®^. Measurements included deviations at rest and during dynamic movement in brow height, smile angle and lower lip position. Facial analysis showed that at rest there was a significant improvement in symmetry in smile angle (*p* = 0.024), and tendency toward better symmetry in brow height and lower lip deviation, however not statistically significant (*p* = 0.365, *p* = 0.240). During dynamic movement there was a significant improvement in symmetry in the upper face (*p* = 0.032), the midface (*p* = 0.005) and the lower face (*p* = 0.042). Details of the pre- and post-treatment values are provided in Figure 2 and Table 2.

### 2.4. Emotion Recognition, FaceReader™

**Figure 3:**
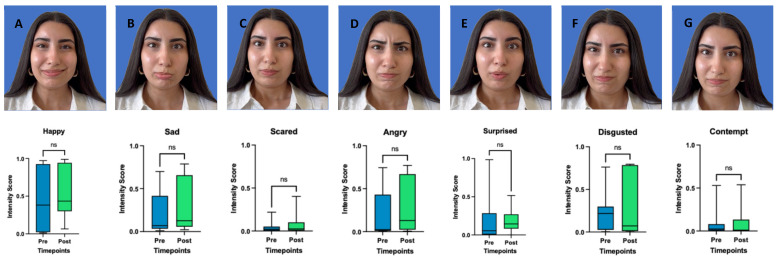
Pictures of each of the seven basic emotions (A = happy, B = Sad, C = Scared, D = Angry, E = Surprised, F = Disgusted, G = Contempt). Intensity Scores before and after (3 weeks) BoNT-A treatment. Boxplots represent the mean score, with whiskers indicating the minimum and maximum values. Statistical significance was assessed using a Wilcoxon matched pairs signed rank test, with a significance threshold set at *p* < 0.05. ns = non-significant.

There was no significant improvement in the ability to express emotions ‘happy’ (*p* = 0.465, CI: −0.2500 to 0.1293), ‘sad’ (*p* = 0.160, CI: −0.2413 to 0.0834), ‘angry’ (*p* = 0.232, CI: −0.3124 to 0.0136), ‘surprised’ (*p* = 0.413, CI: −0.1058 to 0.1293), ‘scared’ (*p* = 0.492, CI: −0.0751 to 0.0338 ‘disgusted’ (*p* = 0.320, CI: −0.3937 to 0.1128), or ‘contempt’ (*p* = 0.232, CI: −0.0226 to 0.0251). See Figure 3.

### 2.5. PROMs

**Figure 4:**
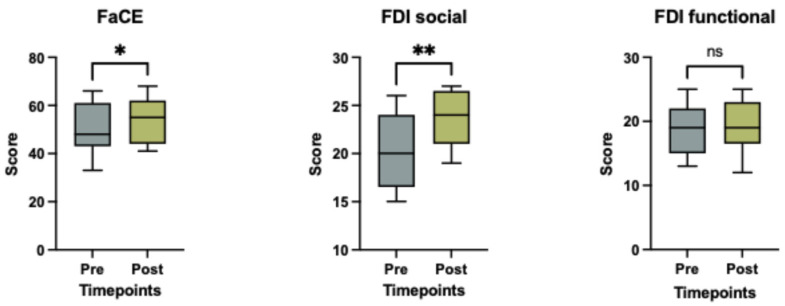
Scores of the FaCE and FDI social and functional questionnaire before and after (3 weeks) BoNT-A treatment. Boxplots represent the mean score, with whiskers indicating the minimum and maximum values. Statistical significance was assessed using a Wilcoxon matched pairs signed rank test, with a significance threshold set at * *p* < 0.05, ** = *p* < 0.005 and ns = non-significant.

Social well-being (FDI, *p* = 0.004) and aesthetic satisfaction (FaCE, *p* = 0.035) significantly improved following treatment, while the functional FDI subscale showed no significant change (*p* = 0.406), as illustrated in Figure 4. There was no correlation between patient-reported outcome measures, including the FDI questionnaire (functional or social well-being score) and the FaCE questionnaire, and the degree of objective facial symmetry improvement after BoNT-A treatment (*p* > 0.05 for all tests).

## 3. Discussion

BoNT-A injections offer a safe, minimally invasive treatment option in non-flaccid facial palsy treatment. Despite its widespread use, the clinical application remains largely guided by individual experience, rather than by standardized, evidence-based protocols [25]. The goal of this study is to investigate how BoNT-A injections impact patients, using both AI-based objective evaluations and PROMs for subjective insights.

**BoNT-A type, dose and adverse effects:** In this study, OnabotulinumtoxinA was administered. The study by Nascimento Remígio et al. [27] demonstrated no significant difference in efficacy between OnabotulinumtoxinA and AbobotulinumtoxinA for the treatment of facial palsy, suggesting that both formulations may be viable options. We injected OnabotulinumtoxinA into the upper face, midface, or lower face, tailored to each patient’s specific needs. In the upper third of the face, injections were directed at the frontalis muscle. In contrast, treatment of the midface and lower third involved targeting the risorius and upper lip elevator muscles. Others prefer targeting the contralateral zygomatic musculature; however, achieving symmetry in these regions is complex. Patients with facial palsy often exhibit a sigmoid smile, where the affected side shows hyperactivity of the depressor anguli oris, pulling the commissure downward and limiting symmetric elevation, while the unaffected side may appear relatively elevated or underactive by contrast. In our hands only small doses are needed to partially weaken the fine facial muscles on the healthy side, as higher doses may overly restrict movement and cause aesthetic or functional issues like lip biting. Our protocol aligns with approaches described by other authors [27] and up to date there are no standardized guidelines in dosing, injections sites, treatment intervals and outcome analysis [25].

The mean total dose of BoNT-A administered in this study was 21 IU (SD 9, range 3–34 IU), which is lower than the doses reported in previous studies, which used an average of 35.2 up to 112.5 IU [28]. Despite the lower dosage, we observed statistically significant improvements in both objective and subjective outcomes. These findings suggest that even a conservative treatment approach can be effective. Starting with a lower dose and adjusting through tailored touch-ups appears to be a safe and effective strategy, allowing treatment to be individualized to the patient’s specific needs.

Follow-up was scheduled three weeks post-injection, aligning with the methodology used in other papers [29,30]. Interestingly, further research indicates that BoNT-A injections remain evident even six months after treatment [27]. This long-term effect has also been observed in animal models, where buccal-to-buccal nerve anastomosis combined with contralateral BoNT-An injections resulted in significantly better innervation than nerve anastomosis alone [31]. These findings suggest that BoNT-A not only suppresses excessive movement on the unaffected side but may also enhance neuromuscular function on the paralyzed side [27]. A short- and long-term follow-up, such as an assessment before the next injection at six months, would be both valuable and interesting.

Physical training after BoNT-A injection was not incorporated in our patients, due to better comparability of the BoNT-A treatment. However, there are reports that it is highly effective when combined with physical therapy, as targeted training helps patients develop relatively normal movement patterns while minimizing synkinetic interference [28]. No complications or adverse effects were observed. BoNT-A has a well-established safety profile, with a low risk of nerve damage, scarring, or extended recovery time. Reported adverse effects in the literature include the following: Temporary muscle weakness (5–15%), typically due to unintended diffusion to adjacent muscles. Mild ptosis (0.5–5%), often resulting from toxin spread. Asymmetry or unintended paralysis (<5%), which resolves as BoNT-A effects wear off [24,32]. Rare systemic reactions, such as difficulty swallowing or speaking, have been reported particularly at higher doses. The literature consistently supports BoNT-A as a safe and effective therapeutic option for restoring facial symmetry in both chronic and acute facial palsy cases, with adverse effects being rare and typically mild [24].

**Objective Symmetry outcome, Emotrics^®^:** Symmetry was assessed with Emotrics^®^. AI-based objective outcome analysis Emotrics^®^ has been validated as an AI-based tool for objective facial symmetry assessment, particularly for evaluating post-interventional changes in facial palsy treatments [19,33]. By providing standardized, landmark-based measurements, Emotrics^®^ enhances treatment evaluation accuracy and an alternative to clinician-led assessments, which are often subject to interobserver variability. In our cohort Emotrics^®^ could successfully be integrated. However, it needed manual landmark adjustments, which possibly could be improved with larger training of the dataset. Our findings demonstrated a significant improvement in facial symmetry. Specifically, dynamic facial expressions revealed statistically significant improvements in symmetry across all three facial regions: upper face (brow height deviation, *p* = 0.032), midface (smile angle, *p* = 0.011), and lower face (lower lip height, *p* = 0.042). While improvements at rest were less pronounced, a significant change in smile angle symmetry (*p* = 0.024) was noted, and trends toward improved brow height and lower lip symmetry were observed, although they did not reach statistical significance. Previous studies have also reported improved facial symmetry following BoNT-A therapy, albeit using a variety of outcome measurement techniques [9]. For example, objective assessments have included marginal reflex distance (MRD) and vertical palpebral distance, as described by Borodic et al. [29]. However, these manual measurement methods are time-consuming and prone to errors. To date, only one study has used Emotrics^®^ to objectively assess the impact of BoNT-A injections in facial palsy patients after receiving free functional gracilis transfer [30]. They concluded that BoNT-A is a safe and feasible intervention for improving asymmetric smiles, suggesting its applicability to a broader patient population. In our hands, the implementation of Emotrics^®^ allowed for an objective and reproducible assessment of facial symmetry. While the software automatically identified key landmarks, manual corrections were occasionally necessary. Optimizing this process could substantially improve workflow efficiency and data reliability.

**Emotion recognition, FaceReader™:** FaceReader™ demonstrated an excellent automatic landmark detection and data processing. However, our findings did not demonstrate statistically significant improvements in the emotions ‘happy’, ‘sad’, ‘angry’, ‘surprised’, ‘scared’, ‘disgusted’, and ‘contempt’ between pre- and post-treatment assessments. One possible explanation lies in the way emotional expressions are quantified in FaceReader™: the emotion scores are computed based on combinations of Action Units (AUs) representing specific muscle activations. While BoNT-A improves facial symmetry, which could theoretically support clearer emotional expressions, it simultaneously reduces muscle activity in the treated areas, which reduces emotion recognition by the software. Thus, a more symmetrical but less active face may result in no improvement in detected emotion scores. Therefore, FaceReader™ showed limited clinical utility in this study. Nevertheless, the tool may hold greater potential in the context of dynamic facial reanimation or face transplantation, where muscle activity gradually increases over time [21].

To date, only one published study by Nguyen et al. [34] has evaluated the use of FaceReader™ in patients with facial synkinesis following BoNT-A treatment. That study found some correlation between FaceReader™ output and eFACE scores but concluded that AI-driven emotion analysis is not yet a reliable tool, which is similar to our findings. A key distinction between their methodology and ours is the way emotions were elicited and analyzed. Nguyen et al. assessed emotional expression through smiling with exposed teeth and analyzed the resulting emotion scores. In contrast, our study employed standardized evoked expressions of all seven emotions. These differing methodologies highlight the complexity and flexibility in applying FaceReader™ to clinical populations. To ensure comparability across studies, we support the establishment of a standardized protocol, as proposed in Müller et al. [35], for using AI-based tools like FaceReader™ in facial palsy research.

Finally, while our study focused on evoked emotional expressions, it is important to note that spontaneous emotional expressions may provide a more valid representation of emotional function. However, capturing spontaneous emotions requires more complex and time-consuming setups [36]. FaceReader™ was trained on both posed and spontaneous datasets, yet its validation is strongest for detecting happiness [37]. Additional validation is still required for reliably detecting the full range of basic emotions [22].

**PROMS:** The physical function subscale of the FDI questionnaire showed no statistically significant difference between pre- and post-treatment assessments (*p* = 0.406). This indicates that patients did not perceive noticeable changes in speaking, eating, or eye closure. However, the social/well-being subscale of the FDI questionnaire showed statistically significant improvement following BoNT-A injection (0.004), reflecting enhanced social confidence and emotional well-being. Patients not only reported a higher quality of life, a more symmetrical facial appearance and reduced feelings of embarrassment but also described an overall more relaxed facial state with less synkinesis. These results suggest that BoNT-A not only reduces synkinetic muscle overactivity on the affected side but also promotes a more harmonious overall facial appearance by indirectly modulating contralateral muscle tone. For instance, the reduction in hyperactivity on the injected side may lead to a compensatory downregulation of muscle activity on the untreated side, resulting in a more relaxed and balanced facial expression. Our results are consistent with previous reports in the literature [27], which similarly observed improvements in patient-reported quality of life. The FaCE questionnaire, which focuses more specifically on patient satisfaction with aesthetic facial appearance, also demonstrated a significant post-treatment improvement (*p* = 0.041), further supporting the positive psychosocial and cosmetic impact.

No correlation was found between PROMS and objective improvements in facial symmetry. These findings interestingly suggest that enhanced symmetry, as measured by AI-based tools, may not directly reflect patients’ perceived social or aesthetic well-being. Other factors, such as the reduction in facial tightness, cramping, discomfort, or synkinesis, may contribute more substantially to subjective satisfaction and quality of life.

**Limitations**: This study has several limitations. The small sample size reduces statistical power and generalizability. Variability in injection sites and doses based on the injector’s assessment and combinations, along with a heterogeneous patient cohort, may have influenced treatment effects. Although the follow-up period of 3 weeks aligns with comparable studies, it remains relatively short and does not allow for assessment of long-term treatment effects. The mean total dose of 22 IU was relatively low, and higher doses with broader injection protocols might yield greater improvements. Emotrics^®^ requires adjustments, which, while AI-assisted, introduces human variability and increases data processing time. Finally, the lack of a control group limits direct comparisons and definitive conclusions on treatment efficacy.

## 4. Conclusions

This study demonstrated the successful integration of both objective and subjective assessment tools into clinical routine for patients with non-flaccid facial palsy. BoNT-A treatment led to significant improvements in facial symmetry, as quantified by Emotrics^®^, and was associated with increased patient-reported satisfaction. However, no measurable improvement in emotional expressivity was detected using FaceReader™. This is likely due to reduced dynamic muscle activity following BoNT-A treatment. Nevertheless, FaceReader™ may prove valuable in dynamic facial reanimation, where muscle activation progressively increases over time. Future studies with larger, more homogeneous samples, control groups, and standardized treatment protocols are needed to validate these findings.

## 5. Materials and Methods

**Study Design:** This prospective observational study was conducted at the University Hospital Basel and Cantonal Hospital of Aarau in 2024. It adhered to the legal regulations of the Swiss Human Research Act and was approved by the local ethics committee (EKNZ 2023-02002). Adult patients with non-flaccid facial palsy who provided informed consent and received BoNT-A injections were included. BoNT-A injections were administered according to standard guidelines, without randomization, and tailored to individual needs. In the upper face, the frontalis muscle was targeted, while in the midface and lower third, injections were directed at the risorius and upper lip elevator muscles. Exclusion criteria included patients with a known allergy to BoNT-A, those under 18 years of age, and individuals without follow-up data. Standardized photographs and videos were taken before and 3 weeks after BoNT-A injections following the guidelines of Santosa et al. [38], ensuring a minimum resolution of 640 × 480 pixels, uniform lighting, and a neutral background. Emotrics^®^ (Massachusetts Eye and Ear Infirmary, Boston, MA, USA) detected automatically 68 landmarks, which were manually reviewed and adjusted if necessary to ensure accuracy. Facial symmetry analysis with Emotrics^®^ was conducted using specific facial expressions for (1) the upper face: photographs at rest and during eyebrow raising assessing the eyebrow deviation (mm), for (2) the midface: photographs at rest and smiling with teeth visible observing the smile angle (degree) and for (3) the lower face: photographs at rest and showing lower teeth assessing the lower lip height deviation (mm). FaceReader™ (version 8.1; Noldus Information Technology, Wageningen, The Netherlands) was used to evaluate emotion recognition, analyzing seven emotions: happiness, sadness, anger, surprise, fear, disgust and contempt. Patients were instructed to evoke each emotion, and the system measured the highest intensity of each expression, reported as a percentage. Additionally, PROMs were assessed at baseline and three weeks post-treatment using the Facial Disability Index (FDI), which includes both functional and social well-being subscales [11]. The FaCE questionnaire was also administered to evaluate patient satisfaction with facial appearance [12]. The primary outcome was objective AI-based assessment of improvements in facial symmetry and emotion recognition combined with the subjective evaluation using PROMs.

**Statistical Analysis** All data were collected and managed using REDCap (Research Electronic Data Capture, Version 9.10, Vanderbilt University) and analyzed with GraphPad Prism (GraphPad Software Inc., Version 10.6.1, San Diego, CA, USA). Continuous variables with a normal distribution are presented as mean ± standard deviation, while non-normally distributed data are reported as median and interquartile range (IQR). Normality was assessed using Q-Q plot inspection and confirmed with the Shapiro–Wilk test. Wilcoxon matched-pairs signed-rank tests were applied for nonparametric data. A *p*-value < 0.05 was considered statistically significant.

## Figures and Tables

**Table 1:** Patient characteristics and etiology of non-flaccid facial palsy patients receiving BoNT-A.

Characteristic	Value
Mean age, years (SD, range)	50.1 (18, 24.5–84.9)
Male/Female sex	1/10
Median BMI, kg/m^2^ (IQR, range)	23.1 (22.3–26.95, 18–38.6)
Smoking	2
Facial palsy etiology	5 Idiopathic 3 Herpes zoster 1 Iatrogenic, 1 Postpartum 1 Tumor

**Table 2:** Facial symmetry measurements before and after Botulinum toxin injection across upper, mid, and lower facial regions. Values shown are medians 95% CI. Statistical significance assessed using the Wilcoxon signed-rank test.

Region	Condition	Timepoint	Photo	Median	95% CI	*p*-Value
Upper Face	Brow height difference (mm)	Before	Rest	2.31 mm	1.057 to 1.90	0.365
		After	Rest	1.33 mm		
		Before	Brows elevated	3.32 mm	0.07 to 2.44	0.032
		After	Brows elevated	2.51 mm		
Mid Face	Smile angle deviation (degree)	Before	Rest	3.11 mm	0.12 to 3.34	0.024
		After	Rest	3.42 mm		
		Before	Smiling showing teeth	11.11 mm	1.06 to 8.69	0.005
		After	Smiling showing teeth	3.12 mm		
Lower Face	Lower lip height difference (mm)	Before	Rest	1.07 mm	−0.459 to 1.80	0.240
		After	Rest	0.78 mm		
		Before	Showing lower teeth	3.76 mm	0.06 to 3.32	0.042
		After	Showing lower teeth	2.38 mm		

## Data Availability

The data presented in this study are not publicly available due to ethical and privacy regulations concerning patient confidentiality. Access to the data is restricted in accordance with institutional and ethical guidelines.

## Abbreviations

The following abbreviations are used in this manuscript:

AI	Artificial Intelligence
BoNT-A	Botulinum toxin Type A
eFACE	Electronic Facial Clinimetric Evaluation
FACE-Q	Facial Aesthetic and Cosmetic Questionnaire
FDI	Facial Disability Index
GCP	Good Clinical Practice
HBGS	House–Brackmann Grading Scale
IQR	Interquartile Range
IU	Injection Units
PROMs	Patient-Reported Outcome Measures
SD	Standard Deviation

## References

[1] Hohman M.H., Hadlock T.A. (2014). Etiology, diagnosis, and management of facial palsy: 2000 patients at a facial nerve center. Laryngoscope.

[2] Moncaliano M.C., Ding P., Goshe J.M., Genther D.J., Ciolek P.J., Byrne P.J. (2023). Clinical features, evaluation, and management of ophthalmic complications of facial paralysis: A review. J. Plast. Reconstr. Aesthet. Surg..

[3] Shamil E., Noriega M., Moin S., Ko T.K., Tan D.J.Y., Meller C., Andrews P., Lekakis G. (2024). Psychological Aspects of Facial Palsy. Facial Plast. Surg..

[4] Hotton M., Huggons E., Hamlet C., Shore D., Johnson D., Norris J.H., Kilcoyne S., Dalton L. (2020). The psychosocial impact of facial palsy: A systematic review. Br. J. Health Psychol..

[5] Nellis J.C., Ishii M., Byrne P.J., Boahene K.D.O., Dey J.K., Ishii L.E. (2017). Association Among Facial Paralysis, Depression, and Quality of Life in Facial Plastic Surgery Patients. JAMA Facial Plast. Surg..

[6] Guntinas-Lichius O., Prengel J., Cohen O., Mäkitie A.A., Vander Poorten V., Ronen O., Shaha A., Ferlito A. (2022). Pathogenesis, diagnosis and therapy of facial synkinesis: A systematic review and clinical practice recommendations by the international head and neck scientific group. Front. Neurol..

[7] Miller M.Q., Hadlock T.A. (2020). Beyond Botox: Contemporary Management of Nonflaccid Facial Palsy. Facial Plast. Surg. Aesthet. Med..

[8] Filipo R., Spahiu I., Covelli E., Nicastri M., Bertoli G.A. (2012). Botulinum toxin in the treatment of facial synkinesis and hyperkinesis. Laryngoscope.

[9] de Sanctis Pecora C., Shitara D. (2021). Botulinum Toxin Type A to Improve Facial Symmetry in Facial Palsy: A Practical Guideline and Clinical Experience. Toxins.

[10] Kahn J.B., Gliklich R.E., Boyev K.P., Stewart M.G., Metson R.B., McKenna M.J. (2001). Validation of a patient-graded instrument for facial nerve paralysis: The FaCE scale. Laryngoscope.

[11] VanSwearingen J.M., Brach J.S. (1996). The Facial Disability Index: Reliability and validity of a disability assessment instrument for disorders of the facial neuromuscular system. Phys. Ther..

[12] Longmire N.M., Wong Riff K.W.Y., O’Hara J.L., Aggarwala S., Allen G.C., Bulstrode N.W., Forrest C.R., French B.M., Goodacre T.E., Marucci D. (2017). Development of a New Module of the FACE-Q for Children and Young Adults with Diverse Conditions Associated with Visible and/or Functional Facial Differences. Facial Plast. Surg..

[13] House J.W., Brackmann D.E. (1985). Facial nerve grading system. Otolaryngol. Head. Neck Surg..

[14] Ross B.G., Fradet G., Nedzelski J.M. (1996). Development of a sensitive clinical facial grading system. Otolaryngol. Head. Neck Surg..

[15] Coulson S.E., Croxson G.R., Adams R.D., O’Dwyer N.J. (2005). Reliability of the “Sydney,” “Sunnybrook,” and “House Brackmann” facial grading systems to assess voluntary movement and synkinesis after facial nerve paralysis. Otolaryngol. Head. Neck Surg..

[16] Banks C.A., Bhama P.K., Park J., Hadlock C.R., Hadlock T.A. (2015). Clinician-Graded Electronic Facial Paralysis Assessment: The eFACE. Plast. Reconstr. Surg..

[17] Boochoon K., Mottaghi A., Aziz A., Pepper J.P. (2023). Deep Learning for the Assessment of Facial Nerve Palsy: Opportunities and Challenges. Facial Plast. Surg..

[18] Hadlock T.A., Urban L.S. (2012). Toward a universal, automated facial measurement tool in facial reanimation. Arch. Facial Plast. Surg..

[19] Guarin D.L., Dusseldorp J., Hadlock T.A., Jowett N. (2018). A Machine Learning Approach for Automated Facial Measurements in Facial Palsy. JAMA Facial Plast. Surg..

[20] Kuilenburg H.V., Uyl MJd (2005). The FaceReader™: Online facial expression recognition. Proceedings of Measuring Behavior.

[21] Dorante M.I., Kollar B., Obed D., Haug V., Fischer S., Pomahac B. (2020). Recognizing Emotional Expression as an Outcome Measure After Face Transplant. JAMA Netw. Open.

[22] Kollar B., Schneider L., Horner V.K., Zeller J., Fricke M., Brugger Z., Gentz M., Kiefer J., Eisenhardt S.U. (2022). Artificial Intelligence-Driven Video Analysis for Novel Outcome Measures After Smile Reanimation Surgery. Facial Plast. Surg. Aesthet. Med..

[23] Boonipat T., Asaad M., Lin J., Glass G.E., Mardini S., Stotland M. (2020). Using Artificial Intelligence to Measure Facial Expression following Facial Reanimation Surgery. Plast. Reconstr. Surg..

[24] Witmanowski H., Błochowiak K. (2020). The whole truth about botulinum toxin—A review. Postepy Dermatol. Alergol..

[25] Cooper L., Lui M., Nduka C. (2017). Botulinum toxin treatment for facial palsy: A systematic review. J. Plast. Reconstr. Aesthet. Surg..

[26] de Jongh F.W., Schaeffers A.W.M.A., Kooreman Z.E., Ingels K.J.A.O., van Heerbeek N., Beurskens C., Monstrey S.J., Pouwels S. (2023). Botulinum toxin A treatment in facial palsy synkinesis: A systematic review and meta-analysis. Eur. Arch. Otorhinolaryngol..

[27] do Nascimento Remigio A.F., Salles A.G., de Faria J.C.M., Ferreira M.C. (2015). Comparison of the efficacy of onabotulinumtoxinA and abobotulinumtoxinA at the 1:3 conversion ratio for the treatment of asymmetry after long-term facial paralysis. Plast. Reconstr. Surg..

[28] Monini S., De Carlo A., Biagini M., Buffoni A., Volpini L., Lazzarino A.I., Barbara M. (2011). Combined protocol for treatment of secondary effects from facial nerve palsy. Acta Otolaryngol..

[29] Borodic G., Bartley M., Slattery W., Glasscock M., Johnson E., Malazio C., Goodnough M., Acquadro M., McKenna M. (2005). Botulinum toxin for aberrant facial nerve regeneration: Double-blind, placebo-controlled trial using subjective endpoints. Plast. Reconstr. Surg..

[30] Ferri A., Zito F., Menapace G., Zannoni C., Bergonzani M., Perlangeli G., Bianchi B. (2023). Optimizing the results of facial animation surgery: Botulinum toxin injection into free functional gracilis flap transfer. J. Plast. Reconstr. Aesthetic Surg..

[31] Guntinas-Lichius O., Glowka T.R., Angelov D.N., Irintchev A., Neiss W.F. (2011). Improved functional recovery after facial nerve reconstruction by temporary denervation of the contralateral mimic musculature with botulinum toxin in rats. Neurorehabil. Neural Repair.

[32] Sethi N., Singh S., DeBoulle K., Rahman E. (2021). A Review of Complications Due to the Use of Botulinum Toxin A for Cosmetic Indications. Aesthetic Plast. Surg..

[33] Guarin D.L., Yunusova Y., Taati B., Dusseldorp J.R., Mohan S., Tavares J., van Veen M.M., Fortier E., Hadlock T.A., Jowett N. (2020). Toward an Automatic System for Computer-Aided Assessment in Facial Palsy. Facial Plast. Surg. Aesthet. Med..

[34] Nguyen C.T., Kollar B., Weber J., Eisenhardt S.U., Weiss J.B.W. (2025). Assessment of Facial Synkinesis Treatment with Botulinum Toxin Using Automated Analysis of Facial Expression: A Pilot Study. Facial Plast. Surg. Aesthet. Med..

[35] Müller S.L.C., Pfister P., Menzi N., Muller L., Klein H.J., Schweizer R., Lee Z.-H., Kollar B., Eisenhardt S.U., Schaefer D.J. (2025). Artificial Intelligence in Facial Palsy Treatment: A Systematic Review and Recommendations. Plast. Reconstr. Surg..

[36] Iacolucci C.M., Banks C., Jowett N., Kozin E.D., Bhama P.K., Barbara M., Hadlock T.A. (2015). Development and validation of a spontaneous smile assay. JAMA Facial Plast. Surg..

[37] Bishay M., Preston K., Strafuss M., Page G., Turcot J., Mavadati M. AFFDEX 2.0: A Real-Time Facial Expression Analysis Toolkit. Proceedings of the 2023 IEEE 17th International Conference on Automatic Face and Gesture Recognition (FG).

[38] Santosa K.B., Fattah A., Gavilán J., Hadlock T.A., Snyder-Warwick A.K. (2017). Photographic Standards for Patients With Facial Palsy and Recommendations by Members of the Sir Charles Bell Society. JAMA Facial Plast. Surg..

